# Fatty liver index predicts incident risk of prediabetes, type 2 diabetes and non-alcoholic fatty liver disease (NAFLD)

**DOI:** 10.1080/07853890.2021.1956685

**Published:** 2021-07-26

**Authors:** Daniel J. Cuthbertson, Juha Koskinen, Emily Brown, Costan G. Magnussen, Nina Hutri-Kähönen, Matthew Sabin, Päivi Tossavainen, Eero Jokinen, Tomi Laitinen, Jorma Viikari, Olli T. Raitakari, Markus Juonala

**Affiliations:** a Metabolism and Nutrition Research Group, Institute of Ageing and Chronic Disease, University of Liverpool, Liverpool, UK; b Research Centre of Applied and Preventive Cardiovascular Medicine, University of Turku, Turku, Finland; c Heart Center, Kotka Central Hospital, Kotka, Finland; d Menzies Institute for Medical Research, University of Tasmania, Hobart, Australia; e Department of Pediatrics, Tampere University and Tampere University Hospital, Tampere, Finland; f Murdoch Children’s Research Institute, The Royal Children’s Hospital and University of Melbourne, Melbourne, Australia; g Department of Pediatrics, PEDEGO Research Unit and Medical Research Center, Oulu University Hospital and University of Oulu, Oulu, Finland; h Department of Pediatric Cardiology, Hospital for Children and Adolescents, University of Helsinki, Helsinki, Finland; i Department of Clinical Physiology and Nuclear Medicine, Kuopio University Hospital, University of Eastern Finland, Kuopio, Finland; j Department of Medicine, University of Turku, Turku, Finland; k Division of Medicine, Turku University Hospital, Turku, Finland

**Keywords:** Non-alcoholic fatty liver disease, metabolic syndrome, obesity, risk, type 2 diabetes

## Abstract

**Aims:**

To investigate the association between overweight/obesity and fatty liver index (FLI) on the odds of incident prediabetes/type 2 diabetes and non-alcoholic fatty liver disease (NAFLD) in 2020 participants after 10 years follow up.

**Methods:**

At baseline (in 2001) 2020 participants, males and females, aged 24–39 years, were stratified according to body mass index (BMI), normal weight (<25 kg/m^2^), overweight (≥25–<30 kg/m^2^), or obese (≥30 kg/m^2^) and FLI (as high FLI ≥60 or low FLI <60). We examined the incidence of prediabetes/type 2 diabetes and NAFLD (ultrasound assessed) over 10 years to 2011 to determine the relative impact of FLI and BMI.

**Results:**

514 and 52 individuals developed prediabetes and type 2 diabetes during follow-up. Such individuals were older, with higher BMI, serum glucose, insulin, alanine aminotransferase (ALT) and triglyceride (TG) concentrations than those who did not develop prediabetes or type 2 diabetes (*n* = 1454). The additional presence of high FLI significantly increased the risk of developing prediabetes and type 2 diabetes above the risk of being overweight/obese. Compared with normal weight, low FLI participants, the odds of prediabetes were ∼2-fold higher and the odds of type 2 diabetes were 9–10-fold higher respectively in the overweight/obese, high FLI group. No difference was observed between normal weight, low FLI and overweight/obese and low FLI groups.

**Conclusions:**

An increased FLI significantly increases the odds of incident prediabetes, type 2 diabetes and NAFLD in individuals with overweight/obese highlighting the contributory role of liver fat accumulation in the pathophysiology of prediabetes/type 2 diabetes.

**Key messages**
- Obesity is a risk factor for non-alcoholic fatty liver disease (NAFLD), prediabetes and type 2 diabetes.
- Additionally, NAFLD is more prevalent in people with prediabetes and type 2 diabetes when compared to age- and BMI-matched individuals.
- The presence of a raised fatty liver index (FLI) confers a significantly increased risk of developing prediabetes, type 2 diabetes and NAFLD above that conferred by being overweight/obese.
- The degree of elevation of FLI can risk stratify for incident prediabetes and type 2 diabetes in people with obesity.

## Introduction

Obesity is the major risk factor for the development of type 2 diabetes with the risk of type 2 diabetes increasing exponentially with body mass index (BMI), a relationship particularly more pronounced in females [1,2]. Additionally, obesity also represents a major risk factor for development of non-alcoholic fatty liver disease (NAFLD) [3]. There is a clear bidirectional relationship between NAFLD and type 2 diabetes with a higher prevalence of NAFLD (both liver steatosis and fibrosis) in type 2 diabetes compared with age- and BMI-matched individuals [4,5] while the prevalence of type 2 diabetes among those with NAFLD and non-alcoholic steatohepatitis (NASH) is estimated to be 22.5% and 43.6%, respectively [6].

Marchesini et al. first suggested NAFLD represented the hepatic manifestation of the metabolic syndrome based on metabolic studies demonstrating hepatic and peripheral insulin resistance in 30 people with biopsy-proven NAFLD, compared with 10 healthy controls and 10 people with type 2 diabetes [7]. However, the contribution of fatty liver to incident type 2 diabetes was first recognized in Pima Indians when high ALT (a biochemical surrogate of NAFLD), associated with increased hepatic glucose output and hepatic insulin resistance, was found to predict development of type 2 diabetes [8]. In the West of Scotland Coronary Prevention Study (WOSCOPS), serum ALT, but not serum aspartate transaminase (AST) levels, increased progressively as the number of metabolic syndrome components increased [9]. The association of serum ALT with metabolic syndrome/incident type 2 diabetes were reproduced in other studies [9–11]. Remarkably, even ALT levels within the upper normal range were associated with higher rates of incident type 2 diabetes [9] while normal serum ALT concentrations do not exclude the presence of NAFLD [4] leading to proposals to revise the normal reference range of serum ALT concentrations [12]. Serum ALT concentration, even with a normal range between 6 and 40 IU/L, has a linear dose-response relationship with risk of metabolic syndrome [13]. A systematic review of all the studies that have assessed the association between ALT level and type 2 diabetes, demonstrated that per 5 IU/L increase in serum ALT, there was a 16% increased risk of incident type 2 diabetes [10].

The strongest evidence of the association between NAFLD and incident type 2 diabetes comes from a meta-analysis of 19 observational cohort studies involving 296,439 people of whom 30.1% had NAFLD, diagnosed by imaging studies [14]. Over a median follow up of 5 years and 16,000 cases of incident type 2 diabetes, NAFLD at baseline associated with a more than doubling of the hazard ratio (HR) of incident type 2 diabetes. Furthermore, the incidence of type 2 diabetes increased with the severity of steatosis (graded by ultrasonography) and in those with advanced fibrosis (higher NAFLD fibrosis scores) at baseline.

One issue that remains contentious is the relative degree by which obesity and NAFLD independently contribute to type 2 diabetes. A recent meta-analysis of participants classified as having metabolically healthy obesity (MHO) *vs*. metabolically unhealthy obesity (MUO) found the risk for incident type 2 diabetes was approximately 4- and 9-fold higher compared with those classified as MHO and normal weight [15], suggesting type 2 diabetes is influenced by the metabolic sequelae of excess weight rather than simply by obesity *per se.*

Previously, Bedogni et al. used data from the Dionysos Nutrition & Liver Study to develop a simple algorithm (based on BMI, waist circumference, serum triglycerides (TGs) and gamma-glutamyl transferase [GGT]), for the prediction of fatty liver/hepatic steatosis (as detected by ultrasonography) in the general population. The algorithm, referred to as the fatty liver index (FLI), had a receiver operating characteristics area under the curve (ROC-AUC) of 0.85 (95% CI 0.81–0.88). Using FLI <30 it can be used to rule out hepatic steatosis (sensitivity of 87%) or to rule in hepatic steatosis when FLI ≥60 (specificity of 86%) for purposes of screening, identifying individuals for intensified lifestyle counselling or in epidemiologic studies [16]. We subsequently validated the FLI as a measure of hepatic steatosis with liver fat determined quantitatively by proton magnetic resonance spectroscopy (^1^H-MRS) [17]. Significantly, the close relationship of NAFLD with insulin resistance and its cardiometabolic burden since childhood is important to consider as the early seeds of disease are planted much earlier in life [18–20]. Therefore, we aimed to examine the relationship between baseline FLI, a non-invasive steatosis score, and overweight/obesity status on the incidence of prediabetes/type 2 diabetes and NAFLD in a cohort of 2020 young adults over a follow up of 10 years.

## Methods

### Participants and study design

The Cardiovascular Risk in Young Finns Study is an ongoing multicentre study examining precursors of atherosclerosis in Finnish children and adolescents. The first cross-sectional survey was conducted in 1980 when 3596 children and adolescents (age 3–18 years) participated [21]. Thereafter, several follow-up studies have been performed with the most recent concluding in 2011.

**Figure 1.**
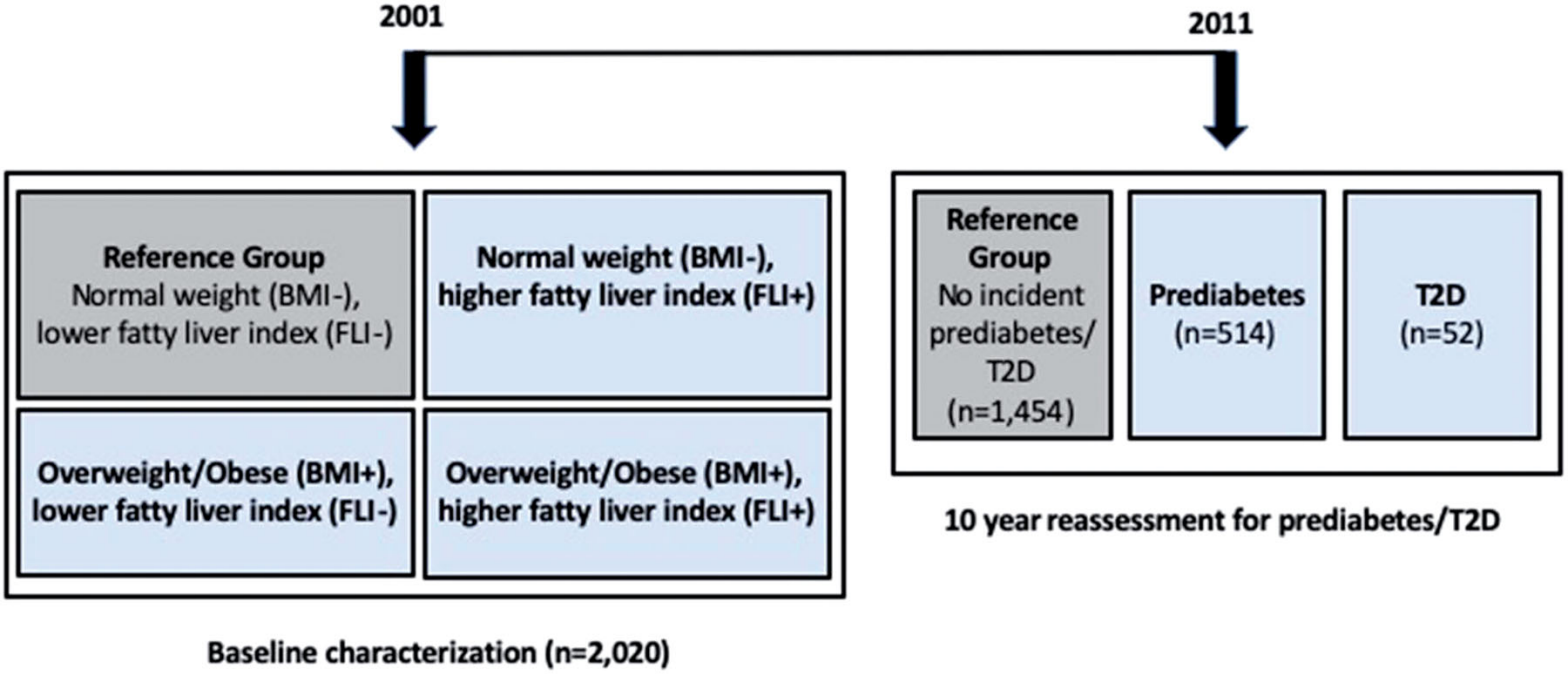
Schematic of study design.

The sample for this analysis included those participants who had ALT and GGT levels measured from the 2001 follow-up study (age 24–39 years) and had participated in the latest follow-up in 2011 (age 34–49 years). A total of 2020, males and females, were included in this analysis (Figure 1). Subjects with type 1 diabetes mellitus (*n* = 12), type 2 diabetes at baseline (*n* = 9) and those female participants who were pregnant at either time point (*n* = 50) were excluded. Participants gave written informed consent and the study was approved by local ethics committees.

*Body composition, blood pressure and questionnaires*: Height, weight and waist circumferences were measured. BMI was calculated using the formula: weight [kg]/(height [m])^2^. Blood pressure was measured using a random zero sphygmomanometer. The average of three measurements was used in the analysis. Smoking habits and alcohol intake was inquired with the use of questionnaires. Subjects were asked to report their alcohol consumption of cans or bottles (1/3 l) of beer, glasses (12 cl) of wine and shots (4 cl) of strong alcohol per week. The values of different beverages consumed during the week allowed us to determine total alcohol intake in grams per day.

*Biomarker measurements*: Venous blood samples were collected after a 12-h fast from the right antecubital vein. Determination of serum glucose and TG concentrations were determined enzymatically (Olympus System Reagent; Olympus System Reagent; Olympus Diagnostica GmbH, Hamburg, Germany) in a clinical analyser (AU400, Olympus, Tokyo, Japan). Serum ALT and GGT activities were measured enzymatically on an automatic analyser (AU400, Olympus, Tokyo, Japan). The mean inter-assay coefficient of variation was 3.7% for ALT and 2.1% for GGT. Serum insulin concentration was determined by a microparticle enzyme immunoassay (IMx insulin reagent, Abbott Diagnostics, Abbott Park, IL) on an IMx instrument (Abbott), with the result used to calculate HOMA-IR [22].

Participants were classified as having prediabetes if they had a glucose 5.6–6.9 mmol/L (100–125 mg/dL) or had a glycated haemoglobin (A1C) level of 5.7–6.4% (42–47 mmol/mol) and reported not receiving oral hypoglycaemic agents and/or insulin injections and did not have type 1 or 2 diabetes; or reported a history of physician-diagnosed type 2 diabetes. Participants were classified as having type 2 diabetes if they had a fasting plasma glucose ≥7.0 mmol/L (≥126 mg/dL), had a glycated haemoglobin (A1C) level of ≥6.5% (48 mmol/mol), reported receiving oral hypoglycaemic agents and/or insulin injections and did not have type 1 diabetes; or reported a history of physician-diagnosed type 2 diabetes.

*Definition of the FLI:* It was determined using an algorithm introduced by Bedogni et al. as follows:

$$({\text{e}}^{0.953*\text{loge}\;(\text{TGs})+0.139*\text{BMI}+0.718*\text{loge}\;\left(\text{GGT}\right)+0.053*\text{waist circumference}-15.745})/(\text{1 }+{\text{ e}}^{0.953*\text{loge}\;(\text{TGs})+0.139*BMI+0.718*loge\;\;\left(GGT\right)+0.053*waist\;circumference-15.745})*100\;\left(16\right).$$

*Reference groups*: Participants were classified further into groups according to their BMI and FLI status at both time points (2001 and 2011). FLI was categorized as low, <60 (FLI−) or high, ≥60 (FLI+). We performed sensitivity analyses using cut-offs for high FLI as ≥85th, ≥80th and 75th percentiles for FLI-value. Results were essentially similar as in the main analyses. These results are presented in the online supplement (Supplementary Table 1). We undertook separate analyses determining the impact of being either overweight (BMI ≥25 kg/m^2^) or obese (BMI ≥30 kg/m^2^) (BMI+) compared with BMI <25 kg/m^2^ or BMI <30 kg/m^2^ (BMI−), respectively. At this time point (2001), the population studied were younger, leaner and relatively healthy. Thus, the reference group was participants with low BMI (BMI−) and low FLI (FLI−).

*Ultrasound imaging*: Ultrasound studies were performed in 2011 (ultrasound was not performed in 2001) by trained physicians and sonographers following standardized protocols and used to determine the presence or absence of NAFLD, based on hepatic steatosis [23]. Examinations were performed with Acuson Sequioia 512 ultrasound (Acuson, Mountain View, CA). Liver fat was scanned using 4.0-Mhz adult abdominal transducers. A trained sonographer graded the liver fat status from the ultrasonographical images using five widely accepted criteria for fatty liver [24]. The liver-to-kidney contrast, parenchymal brightness, deep beam attenuation, bright vessel walls and visibility of the neck of the gallbladder. For statistical analyses, we used a binary outcome variable (normal *vs.* fatty liver) based on the sonographer’s clinical judgement of the image data.

*Statistical methods*: Values for FLI, serum ALT, GGT and TG, were log_e_-transformed to correct for skewness. Participant characteristics were compared using t-tests and chi-square tests as appropriate. Linear regression adjusted for age and sex was used to assess associations between risk factors and future outcome groups (no pre-diabetes or type 2 diabetes, pre-diabetes and type 2 diabetes). To assess the independent associations between risk factors and type 2 diabetes, we performed stepwise multivariable regression modelling using SAS stepwise selection. In initial models, all variables in univariable model were included. Next, variables were removed from the model one by one until all the variables remaining in the model were statistically significant (*p* < .05). At each step, the variable showing the smallest contribution to the model was removed. Age-and sex-adjusted logistic regression model was used to calculate odds ratios and 95% confidence intervals (CIs). Statistical analyses were performed with SAS version 9.4 (SAS Institute Inc., Cary, NC). Statistical significance was inferred as a *p* value of less than .05.

## Results

*Participant characteristics*: Participant characteristics for all participants are shown in Table 1 stratified by sex and by pre-diabetes/type 2 diabetes status. Significant differences were observed in BMI and in serum glucose, liver enzyme concentrations (serum ALT and GGT) and TG concentrations between sexes (*p* always < .0001). Of the original cohort of 2020 individuals, 514 participants (201 males, 304 females) developed prediabetes and 52 participants (28 males, 24 females) developed incident type 2 diabetes in 2011. As expected, the risk of type 2 diabetes varied according to BMI for each sex (Supplementary Table 2). The baseline clinical characteristics (in 2001) for those who did not develop incident prediabetes/type 2 diabetes and for those who developed either prediabetes or type 2 diabetes were compared. Individuals who developed either prediabetes or type 2 diabetes were older, with higher BMI, waist circumference, serum glucose, insulin and HOMA-IR, liver enzyme concentrations (ALT and GGT), TG concentrations and FLI values. Participants free from incident pre-diabetes and type 2 diabetes also had lower glucose, insulin and HOMA-IR compared to those who did develop type 2 diabetes but not compared to those who developed prediabetes. Results were similar when those excluded were included in the main analysis.

**Table 1.** Participant characteristics at baseline (2001) according to prediabetes or type 2 diabetes (T2D) status at follow-up (2011).

	Male	Female	No incident prediabetes/T2D	Incident prediabetes	Incident T2D	*p* Value for trend
*N*	923 (44%)	1097 (56%)	1454	514	52	–
Age (years)	31.7 (5.0)	31.9 (4.9)	31.6 (5.0)	32.5 (4.9)	34.1 (4.6)	.011
BMI (kg/m^2^)	25.7 (4.0)	24.4 (4.6)	24.5 (4.2)	25.8 (4.3)	31.0 (6.2)	<.0001
Waist circumference (cm)	89.9 (10.7)	79.1 (11.2)	83.6 (12.0)	87.1 (11.7)	98.0 (14.7)	<.0001
ALT (mmol/L)	14.5 (10.3)	8.5 (5.3)	10.5 (8.3)	12.3 (9.2)	17.7 (10.6)	<.0001
GGT (U/L)	32.5 (26.2)	18.0 (12.9)	23.1 (21.0)	26.4 (19.1)	38.7 (24.0)	<.0001
TG (mmol/L)	1.53 (1.02)	1.17 (0.70)	1.28 (0.87)	1.42 (1.05)	1.78 (0.86)	<.0001
FLI (units)	40.1 (5.1–92.7)	19.7 (2.3–76.2)	28.1 (2.8–87.6)	34.9 (3.3–90.2)	64.2 (4.8–98.1)	<.0001
Glucose (mmol/L)	5.18 (0.58)	4.88 (0.45)	5.0 (0.53)	5.02 (0.32)	5.53 (0.48)	<.0001
Insulin (mIU/L)	7.6 (5.0)	7.7 (5.6)	7.2 (4.76)	7.6 (4.8)	14.4 (6.5)	<.0001
HOMA-IR (units)	0.09 (0.07)	0.10 (0.09)	0.09 (0.06)	0.09 (0.06)	0.20 (0.10)	<.0001

ALT: alanine aminotransferase; BMI: body mass index; FLI: fatty liver index (95% confidence intervals); GGT: gamma-glutamyl transferase; HOMA-IR: homeostatic model assessment of insulin resistance; TG: triglycerides.

*p* Value for trend according to prediabetes or T2D status at follow-up using linear regression adjusted for age and sex.

The HOMA-IR was calculated by multiplying fasting Insulin (U/mL) by fasting glucose (mmol/L) and dividing by 22.5.

### Longitudinal associations between risk factors and incident NAFLD and type 2 diabetes

When we examined the association between risk factors in 2001 and risk of either NAFLD or type 2 diabetes in 2011, we found that all risk factors including adiposity markers (waist circumference and BMI) and FLI were associated with ultrasound-diagnosed NAFLD and type 2 diabetes in 10-year follow-up (Table 2). Next, we constructed a multivariable stepwise regression model assessing the independent relation between baseline risk factors and future NAFLD and type 2 diabetes (Table 3). Age, sex and alcohol consumption were forced into the models. The model included FLI, ALT, GGT, waist circumference, BMI and TG.

**Table 2.** Independent association between risk factors in 2001 and non-alcoholic fatty liver disease (NAFLD) and type 2 diabetes (T2D) in 2011.

	NAFLD		T2D	
Risk factor	OR (95% CI*)	*p* Value	OR (95% CI*)	*p* Value
Waist circumference (cm)	1.09 (1.05–1.13)	<.0001	1.17 (1.04–1.32)	.01
BMI	1.23 (1.12–1.34)	<.0001	1.42 (1.09–1.74)	.009
(log)FLI	3.50 (2.16–5.67)	<.0001	1.06 (1.01–1.11)	.017
(log)ALT	6.26 (2.67–14.7)	<.0001	10.2 (1.04–100.2)	.05
(log)GGT	3.86 (1.80–8.26)	<.0001	3.05 (0.31–39.3)	.31
(log)TG	3.15 (1.62–6.15)	<.0001	0.70 (0.06–9.06)	.78

All models include adjustment for age and sex.

*Odds ratios and their 95% confidence intervals (OR 95% CI) are for NAFLD and T2D for a one unit increase in the baseline risk factor.

**Table 3.** Multivariable stepwise logistic regression model assessing the association between risk factors in 2001 and a) NAFLD and b) Type 2 diabetes (T2D) in 2011.

a)					
Continuous	NAFLD		Dichotomous	NAFLD	
risk factor	OR (95% CI)*	*p* Value	risk factor	OR (95% CI)*	*p* Value
(log)FLI	3.09 (2.53–3.78)	<.0001	High FLI	1.73 (1.18–2.53)	.005
(log)ALT	1.50 (1.12–2.02)	.0072	High ALT	1.63 (1.13–2.53)	.009
(log)GGT	**	**	High GGT	1.54 (1.06–2.26)	.026
waist	**	**	High waist	**	**
BMI	**	**	High BMI	2.30 (1.68–3.14)	<.0001
(log)TG	**	**	High TG	2.26 (1.59–3.21)	<.0001

b)					
Continuous	T2D		Dichotomous	T2D	
risk factor	β ± SE	*p* Value	risk factor	β ±SE	*p* Value
(log)FLI	3.84 (2.31–6.38)	<.0001	High FLI	2.67 (1.13–6.27)	.0026
(log)ALT	2.25 (1.28–3.98)	.005	High ALT	**	**
(log)GGT	**	**	High GGT	2.86 (1.49–5.46)	.0015
waist	**	**	High waist	3.48 (1.54–7.58)	.0017
BMI	**	**	High BMI	**	**
(log)TG	**	**	High TG	**	**

All models include adjustment for age, sex and alcohol consumption.

High FLI was determined as having index > = 60 units.

ALT, GGT and triglyceride were determined as ≥ 85 percentile.

High BMI ≥ 25 kg/m^2^, high waist ≥ 88 cm in women and ≥102cm in men.

*Odds ratios and their 95% confidence intervals (OR 95% CI) are for NAFLD and T2D for a one unit increase in the baseline risk factor.

**Variable did not meet the .05 significance level for entry into the model.

*Incident NAFLD* (Table 3(a)): FLI remained associated with increased risk for NAFLD when treated as continuous variables and dichotomous variables (OR [95% CI], 3.09 [2.53–3.78] and 1.73 [1.18–2.53]); similarly, ALT remained associated with increased risk for NAFLD when treated as continuous variables and dichotomous variables (OR [95% CI], 1.50 [1.12–2.02] and 1.63 [1.13–2.53], respectively. High BMI and high TG were additionally associated with future NAFLD when treated as dichotomous variable in a stepwise model. Variables that did not meet the *p* ≤ .05 significance level during the stepwise procedure and thus were dropped from the model. Due to high multicollinearity between risk factors the results should be interpreted cautiously when included in the same model.

*Incident type 2 diabetes* (Table 3(b)): FLI remained associated with increased risk for type 2 diabetes when treated as continuous variables and dichotomous variables (OR [95% CI] 3.84 [2.31–6.38] and 2.67 [1.13–6.27], high ALT remained associated with increased risk for type 2 diabetes when treated as continuous variables (OR 2.25 [95% CI] [1.28–3.98]). Waist circumference, BMI, GGT and TG did not meet the *p* ≤ .05 significance level during the stepwise procedure and thus were dropped from the model.

*Prediction of prediabetes and type 2 diabetes between different phenotypes assessing overweight/obesity and FLI*: Table 4 shows the odds ratio estimates (OR 95% CI) in predicting prediabetes between participants that were normal weight and low-FLI (BMI−, FLI−) (reference group), participants that were normal weight with high-FLI (BMI−, FLI+), overweight but low-FLI (BMI+, FLI−) and participants with overweight/obesity and had a high-FLI (BMI+, FLI+). In normal weight participants, a higher FLI did not significantly increase the risk of prediabetes *vs.* lower FLI participants; however, a higher FLI in participants with overweight and obesity was associated with a significantly higher risk of prediabetes than those with a lower FLI (OR 2.12 [1.57–2.85] and 1.87 [1.28–2.72]).

**Table 4.** Odds ratio (OR) and 95% confidence intervals (CI) for incident prediabetes according to body mass index (BMI) and fatty liver index (FLI) status (FLI + when > = 60 units, FLI − when < 60 units) at baseline a) BMI < 25 kg/m^2^ and b) BMI < 30 kg/m^2^.

a)				
	Frequency (*n* = 2020)	Percent	OR (95% CI)	(*p* Value)
(BMI−, FLI−)	1300/335	64.3	Reference	Reference
(BMI−, FLI+)	10/4	0.5	2.06 (0.50–8.41)	.31
(BMI+, FLI−)	438/162	21.7	1.68 (1.33–2.14)	<.0001
(BMI+, FLI+)	272/124	13.5	2.12 (1.57–2.85)	<.0001

b)				
	Frequency (*n* = 2020)	Percent	OR (95% CI)	(*p* Value)
(BMI−, FLI−)	1703/479	84.3	Reference	Reference
(BMI−, FLI+)	121/64	6.0	1.87 (1.26–2.77)	<.0001
(BMI+, FLI−)	35/18	1.7	4.47 (2.17–9.23)	<.0001
(BMI+, FLI+)	161/64	8.0	1.87 (1.28–2.72)	<.0001

Table 5 shows the odds ratio estimates (OR 95% CI) in predicting type 2 diabetes between participants that were normal weight and low-FLI (BMI−, FLI−) (reference group), participants with overweight/obesity but low-FLI (BMI+, FLI−) and participants with overweight/obesity and had a high-FLI (BMI+, FLI+). Not enough participants with normal weight but high-FLI (BMI−, FLI+) were observed to perform analysis. Normal weight participants with a higher FLI did not increase the risk of developing type 2 diabetes 10 years later. However, a higher FLI in participants with overweight/obesity was associated with a significantly higher risk of type 2 diabetes than those with a lower FLI (OR 10.3 [5.69–18.4] and 8.94 [4.86–16.5]).

**Table 5.** Odds ratio (OR) and 95% confidence intervals (CI) for incident T2D according to body mass index (BMI) and fatty liver index (FLI) status (FLI + when > = 60 units, FLI − when < 60 units) at baseline A) BMI < 25 kg/m^2^ and B) BMI < 30 kg/m^2^.

A)				
	Frequency (*n* = 2020)	Percent	OR (95 % CI)	(*p* Value)
(BMI−, FLI−)	1300/18	64.3	Reference	Reference
(BMI−, FLI+)	10/1	0.5	–	–
(BMI+, FLI−)	438/10	23.7	0.99 (0.42–2.28)	0.92
(BMI+, FLI+)	272/23	13.5	10.3 (5.69–18.4)	<0.0001

B)				
	Frequency (*n* = 2020)	Percent	OR (95% CI)	(*p* Value)
(BMI−, FLI−)	1703/27	84.3	Reference	Reference
(BMI−, FLI+)	121/2	6.0	1.08 (0.25–4.76)	.95
(BMI+, FLI−)	35/1	1.7	1.62 (0.21–12.4)	.56
(BMI+, FLI+)	161/22	8.0	8.94 (4.86–16.5)	<.0001

## Discussion

This study, in a large cohort of 2020 young Finnish men and women, examined the interaction between FLI and being overweight or obese in the subsequent development (over 10 years) of prediabetes, type 2 diabetes and NAFLD. In participants with overweight or obesity, higher FLI values increased the risk of prediabetes more than 3-fold compared with similar BMI-matched individuals with lower FLI; more strikingly higher FLI increased the risk of type 2 diabetes by more than 10- and 15-fold in participants with overweight or obesity compared with similar BMI-matched individuals with lower FLI. This highlights a stratification of risk according to weight category and presence/absence of NAFLD (indicative of liver fat accumulation) for incident prediabetes and type 2 diabetes.

Other studies have explored an association between NAFLD and type 2 diabetes based on biochemical surrogates of NAFLD or through imaging studies, e.g. abdominal ultrasonography. However, we used the FLI as a simple and accurate predictor of hepatic steatosis in the general population. FLI was originally developed using ultrasonography first suggested by Bedogni et al. and we subsequently validated its utility in predicting the likelihood of NAFLD in healthy controls and insulin-resistant individuals with obesity using proton magnetic resonance spectroscopy (^1^H-MRS) [16,17]. The association of higher FLI with type 2 diabetes has been noted in several previous studies [25–27]. First, in the PREDAPS study, examining a Spanish cohort of 1142 adults with prediabetes, of whom 55.7% had hepatic steatosis (FLI > 60), FLI ≥ 60 was independently associated with type 2 diabetes incidence at 3 years of follow up, HR 4.97 (95% CI 2.28–10.8) and 3.21 (95% CI 1.45–7.09) in a fully adjusted model [27]. Second, in another study of 1792 Finnish men examining the associations of fatty liver with incident type 2 diabetes, there was a steady increase in HR of incident type 2 diabetes across FLI categories of 10, with every unit increase in FLI associated with 1.8% increase in HR. Compared with FLI of ≤ 30, an FLI 30− ≤ 60 was associated with HR of 1.79 (1.33, 2.37) and an FLI ≥ 60 with HR of 2.63 (1.89, 3.66) [25]. In a further study of 2784 Korean adults a similar relationship was seen with FLI according to the same groupings of FLI: ≤30, 30− ≤ 60 or ≥ 60: odds ratio for new-onset type 2 diabetes for FLI < 30 *vs.* 30–59 *vs.* ≥ 60 was 1.87 (1.05, 3.33) and 2.84 (1.4, 5.75) [26]. Interestingly a recent study demonstrated that a change in NAFLD status over time, determined by serial FLI and abdominal ultrasonography, influences the risk of incident type 2 diabetes: the risk with resolved NAFLD was not significantly different from that with no NAFLD [28].

This study attempts to gain further insight by examining the synergistic risk between weight status (overweight/obese) and presence/absence of NAFLD. The findings we present concur with findings from a large Korean population of 34,258 participants (without type 2 diabetes) where the influence of NAFLD (implied from a high FLI measurement) as a determinant of incident type 2 diabetes was examined. The risk of incident type 2 diabetes in individuals with obesity who were MHO, with a low FLI, was not significantly increased compared with the MHO non-obese; in contrast, individuals with obesity who were MUO, with a high FLI, had an almost 2-fold risk of incident type 2 diabetes [29]. While MHO is associated with a lower risk of incident type 2 diabetes compared with MUO [30], a meta-analysis of all the studies examining different obesity phenotypes clearly indicate that although being MHO confers some protection, all adults with obesity show a substantially increased risk of developing type 2 diabetes compared with MHO normal-weight adults [15].

So why does NAFLD seem to be associated with type 2 diabetes? Attempts to differentiate healthy and unhealthy normal weight and obesity have focussed on body fat distribution and ectopic fat deposition but have highlighted that poor metabolic health, regardless of weight, is characterized by higher liver fat, higher visceral fat and insulin resistance [31]. The mechanism behind this relationship between liver fat, metabolic syndrome and type 2 diabetes was explored in a study investigating the dose response between liver fat and metabolic end points [32]. Once liver fat accumulation exceeds 6 ± 2%, slightly exceeding the upper limit of normal of liver fat in the general population (5.56%) as defined in the Dallas Heart Study [33], skeletal muscle insulin resistance, hypertriglyceridaemia and low HDL-cholesterol become evident. In addition to steatosis, the association of liver fibrosis with type 2 diabetes has been noted by others. In a retrospective study of 396 patients with biopsy-proven NAFLD, Bjorkstrom et al. found a higher proportion of patients with fibrosis stages 3– develop type 2 diabetes than patients with fibrosis stages 0–2 while in patients with fibrosis stages 0–2, fat score was associated with risk of type 2 diabetes [34].

The limitations of the study include the relatively small number of participants who subsequently developed type 2 diabetes. We also acknowledge the limitation of using a surrogate measure of NAFLD at baseline, the FLI, with no abdominal ultrasonography available although validations studies with ultrasound and even proton-magnetic resonance spectroscopy demonstrates its accuracy. Due to high multicollinearity, results from the regression model including FLI and any of its parameters together, should be interpreted cautiously.

In summary, we find that the degree of elevation of the FLI significantly modulates the risk of incident prediabetes or type 2 diabetes associated with being overweight or obese.

## Supplementary Material

Supplemental Material

## Data Availability

The data that support the findings of this study are available from author MJ, upon reasonable request.
