# Supplementary material for: Fatty liver index predicts incident risk of prediabetes, type 2 diabetes and non-alcoholic fatty liver disease (NAFLD)
Source: Ann Med. 2021 Jul 26;53(1):1257–65. doi: 10.1080/07853890.2021.1956685 (PMC8317942; doi:10.1080/07853890.2021.1956685)
Supplement: Supplemental Material [file IANN_A_1956685_SM7465.docx]

**Online Supplementary Tables**

**Supplementary Table 1** Multivariable stepwise logistic regression model assessing the association between risk factors in 2001 and **a)** NAFLD and **b)** Type 2 diabetes (T2D) in 2011. High FLI defined as **i)** ≥75^th^ percentile and **ii)** ≥80^th^ percentile and **iii)** ≥85^th^ percentile

**i) ≥75^th^ percentile**

**a)**

| Dichotomous | NAFLD |  |
| --- | --- | --- |
| risk factor | OR (95% CI)* | P-value |
| High FLI | 2.28 (1.56-3.32) | <0.0001 |
| High ALT | 1.77 (1.26-2.62) | 0.0010 |
| High GGT | ** | ** |
| High waist | ** | ** |
| High BMI | 1.97 (1.41-2.74) | <0.0001 |
| High trigly | 2.14 (1.50-3.04) | <0.0001 |

**b)**

| Dichotomous | T2D |  |
| --- | --- | --- |
| risk factor | β±SE | P-value |
| High FLI | 2.88 (1.15-7.20) | 0.024 |
| High ALT | 2.64 (1.42-4.93) | 0.0023 |
| High GGT | ** | ** |
| High waist | 2.89 (1.23-6.80) | 0.015 |
| High BMI | ** | ** |
| High TG | ** | ** |

All models include adjustment for age, sex and alcohol consumption.

High FLI, ALT, GGT and triglyceride were determined as ≥ 85 percentile

High BMI ≥ 25 kg/m^2^, high waist ≥ 88cm in women and ≥102cm in men

* Odds ratios and their 95% confidence intervals (OR 95% CI) are for NAFLD and T2D for a one unit increase in the baseline risk factor.

** Variable did not meet the 0.05 significance level for entry into the model

**ii) ≥80^th^ percentile**

**a)**

| Dichotomous | NAFLD |  |
| --- | --- | --- |
| risk factor | OR (95% CI)* | P-value |
| High FLI | 2.28 (1.56-3.32) | <0.0001 |
| High ALT | 1.74 (1.22-2.64) | 0.0015 |
| High GGT | ** | ** |
| High waist | ** | ** |
| High BMI | 2.09 (1.52-2.88) | <0.0001 |
| High trigly | 2.21 (1.56-3.13) | <0.0001 |

**b)**

| Dichotomous | T2D |  |
| --- | --- | --- |
| risk factor | β±SE | P-value |
| High FLI | 3.41 (1.37-8.48) | <0.0001 |
| High ALT | 2.49 (1.32-4.70) | 0.0041 |
| High GGT | ** | ** |
| High waist | 2.59 (1.09-6.14) | 0.030 |
| High BMI | ** | ** |
| High TG | ** | ** |

All models include adjustment for age, sex and alcohol consumption.

High FLI, ALT, GGT and triglyceride were determined as ≥ 85 percentile

High BMI ≥ 25 kg/m^2^, high waist ≥ 88cm in women and ≥102cm in men

* Odds ratios and their 95% confidence intervals (OR 95% CI) are for NAFLD and T2D for a one unit increase in the baseline risk factor.

** Variable did not meet the 0.05 significance level for entry into the model

**iii) ≥85^th^ percentile**

a)

| Dichotomous | NAFLD |  |
| --- | --- | --- |
| risk factor | OR (95% CI)* | P-value |
| High FLI | 2.29 (1.54-3.38) | <0.0001 |
| High ALT | 1.79 (1.27-2.53) | 0.0008 |
| High GGT | ** | ** |
| High waist | ** | ** |
| High BMI | 2.24 (1.65-3.05) | <0.0001 |
| High trigly | 2.22 (1.57-3.16) | <0.0001 |

b)

| Dichotomous | T2D |  |
| --- | --- | --- |
| risk factor | β±SE | P-value |
| High FLI | 9.41 (4.88-18.1) | <0.0001 |
| High ALT | 2.24 (1.18-4.29) | 0.013 |
| High GGT | ** | ** |
| High waist | ** | ** |
| High BMI | ** | ** |
| High TG | ** | ** |

All models include adjustment for age, sex and alcohol consumption.

High FLI, ALT, GGT and triglyceride were determined as ≥ 85 percentile

High BMI ≥ 25 kg/m^2^, high waist ≥ 88cm in women and ≥102cm in men

* Odds ratios and their 95% confidence intervals (OR 95% CI) are for NAFLD and T2D for a one unit increase in the baseline risk factor.

** Variable did not meet the 0.05 significance level for entry into the model

**Supplementary Table 2** Odds ratio (OR) and 95% confidence intervals (CI) of type 2 diabetes according to body mass index (BMI) stratified by sex

|  | **Males** | |  | **Females** | |
| --- | --- | --- | --- | --- | --- |
| **BMI (N)** | **OR (95% CI)** | **P-value** | **BMI (N)** | **OR (95% CI)** | **P-value** |
| <25 kg/m^2^ (768) | ref |  | <25 (542) | ref |  |
| 25-30 kg/m^2^ (232) | 2.4 (0.8-6.9) | 0.12 | 25-30 (282) | 0.7 (0.2-2.1) | 0.21 |
| 30-35 kg/m^2^ (66) | 12.2 (4.4-33.8) | 0.013 | 30-35 (81) | 5.6 (2.2-13.9) | 0.012 |
| >35 kg/m^2^ (31) | 27.9 (9.5-81.7) | <0.001 | >35 (18) | 16.1 (4.8-54.0) | <0.001 |

Age- adjusted multivariable model
